# Flash Glucose Monitoring in Croatia: The Optimal Number of Scans per Day to Achieve Good Glycemic Control in Type 1 Diabetes

**DOI:** 10.3390/medicina59111893

**Published:** 2023-10-25

**Authors:** Silvija Canecki-Varzic, Ivana Prpic-Krizevac, Maja Cigrovski Berkovic, Dario Rahelic, Ema Schonberger, Marina Gradiser, Ines Bilic-Curcic

**Affiliations:** 1 Department of Endocrinology, Clinical Hospital Center Osijek, 31000 Osijek, Croatia; canecki.silvija@kbco.hr (S.C.-V.); ipkrizevac@mefos.hr (I.P.-K.); ema.schonberger@kbco.hr (E.S.); 2 Department of Pathophysiology, Faculty of Medicine, Josip Juraj Strossmayer University of Osijek, 31000 Osijek, Croatia; 3 Department of Internal Medicine and History of Medicine, Faculty of Medicine, Josip Juraj Strossmayer University of Osijek, 31000 Osijek, Croatia; 4 Department of Medicine of Sports and Exercise, Faculty of Kinesiology, University of Zagreb, 10000 Zagreb, Croatia; maja.cigrovski.berkovic@kif.unizg.hr; 5 University Clinic for Diabetes Vuk Vrhovac, 10000 Zagreb, Croatia; dario.rahelic@unicath.hr; 6 School of Medicine, Catholic University of Croatia, 10000 Zagreb, Croatia; 7 Faculty of Medicine, Josip Juraj Strossmayer University of Osijek, 31000 Osijek, Croatia; 8 Department of Internal Medicine, County Hospital Čakovec, 40000 Čakovec, Croatia; marina.gradiser@gmail.com; 9 Department of Pharmacology, Faculty of Medicine, Josip Juraj Strossmayer University of Osijek, Josipa Huttlera 4, 31000 Osijek, Croatia

**Keywords:** continuous glucose monitoring, FreeStyle Libre, blood glucose monitoring frequency, glycemic measures

## Abstract

*Background and Objectives*: The purpose of this study is to determine the optimal number of scans per day required for attaining good glycemic regulation. *Materials and Methods*: The association of scanning frequency and glucometrics was analyzed according to bins of scanning frequency and bins of time in range (TIR) in the Croatian population of type 1 diabetes (T1DM) patients. *Results*: Intermittently scanned continuous glucose monitoring (isCGM) Libre users in Croatia performed on average 13 ± 7.4 scans per day. According to bins of scanning frequency, bin 5 with 11.2 ± 02 daily scans was sufficient for achieving meaningful improvements in glycemic regulation, while decreasing severe hypoglycemia required an increasing number of scans up to bin 10 (31 ± 0.9), yet with no effect on TIR improvement. When data were analyzed according to bins of TIR, an average of 16.3 ± 10.5 scans daily was associated with a TIR of 94.09 ± 3.49% and a coefficient of variation (CV) of 22.97 ± 4.94%. Improvement was shown between each successive bin of TIR but, of notice, the number of scans performed per day was 16.3 ± 10.5 according to TIR-based analysis and 31.9 ± 13.5 in bin 10 according to scan frequency analysis. *Conclusions*: In conclusion, an optimal average number of scans per day is 16.3 in order to achieve glucose stability and to minimize the burden associated with over-scanning.

## 1. Introduction

The number of patients with diabetes is reaching pandemic proportions. In patients with type 1 diabetes (T1DM), as well as in those with type 2 diabetes (T2DM) on multiple daily insulin injections (MDIs), good glycemic regulation is imperative to reduce the risk of acute and chronic complications of diabetes [1]. However, good glycemic regulation and treatment of hyperglycemia in patients on MDI or insulin pumps can at the same time result in hypoglycemia with adverse clinical outcomes [2]. In these patients, frequent glycemic self-monitoring (SMBG) allows for insulin dose adjustments and improved metabolic control. Previous studies have shown that an increasing frequency of SMBG is associated with lower A1C [3]. However, repeated finger pricks are painful and uncomfortable for patients, and somewhat unpractical, with interruption of daily activities [4]. In addition, SMBG has other limitations: it does not show glycemic variability and it cannot always detect episodes of hypoglycemia and hyperglycemia due to too infrequent monitoring [5,6], which may also affect therapeutic decisions leading to therapeutic oversights.

Continuous glucose monitoring (CGM) provides the ability to frequently control glucose concentration in the interstitial fluid and opens new and exciting opportunities in daily diabetes management. Intermittently scanned CGM (isCGM) detects glucose levels every 1–5 min as opposed to real-time CGM (rtCGM), providing data on glucose levels in real-time, which almost eliminates the need for SMBG. Information on glucose fluctuations, trends, and episodes of low and high glucose levels can be obtained with trend arrows and alarms, which significantly improve patient self-management and represent a major advance in the treatment of diabetes [7]. Of the isCGM systems, only the FreeStyle Libre (FSL) 2, recently placed on the market, features alarms for hypo- and hyperglycemia, unlike the rtCGM systems such as Dexcom, which all have this option [8,9]. However, the majority of users, primarily due to health insurance policies, are presently supplied with the isCGM FSL without an alarm option, and the question arises as to whether adequate diabetes management can be achieved with this system, yet escaping excessive scanning, which represents a great burden for people with T1DM.

Furthermore, FSL allows patients to control their glucose levels frequently, without painful finger pricking, but must be accompanied by proactive sensor scanning. FSL was examined in T1DM and T2DM patients in two clinical studies, IMPACT and REPLACE, respectively [10,11]. In patients with T1DM and good diabetes management, there was a clinically significant reduction in the time spent in hypoglycemia during the first 15 days of system use, which persisted for 6 months. In patients with T2DM and poor diabetes regulation, there was also a decrease in the time spent in hypoglycemia, with a decrease in A1C but only in the group of patients younger than 65 years.

isCGM FSL appeared on the EU market in 2014 and is available in Croatia since September 2018. In a previous study, we showed in T1DM patients how 3 months of FSL use led to a significant drop in A1C mainly driven by those patients whose A1C was >7% (53 mmol/mol), and the improvement in A1C was mainly correlated to the increase in the number of scans per day [12].

In the present paper, we expand the results and examine the use of FSL in Croatia from November 2018 to May 2022; firstly, we examined the frequency of monitoring; secondly, we examined the association of glucose metrics and frequency of scanning and time in range (TIR); and, thirdly, we compared our results with the available worldwide data. In addition, given that the previous real-world (RW) studies demonstrated an unequivocal positive correlation between scanning frequency and improvement in glucose metrics, we aimed to determine the optimal number of scans per day required for attaining good glycemic regulation.

## 2. Materials and Methods

### 2.1. Study Population and Data Collection

This was a Croatian nation-based cohort study including patients with T1DM using FSL system. Data were collected from 6211 FSL readers from November 2018 to May 2022 using the LibreView platform; Abbott Diabetes Care, Inc, a complementary software capable of generating reports obtained from the FSL Reader, including data on glucose metrics as defined by the international consensus on TIR [13]. Data gathered by smartphone app were excluded from the analysis. Before data storage, written informed consent was obtained, and patients’ data were changed to avoid disclosing any personal data. Generated anonymous dataset was stored in a cloud database and used for further analysis of Croatian and other published worldwide populations.

### 2.2. Data Analysis

The available data from database sets were gathered, including information on the number of readers and sensors, duration of isCGM monitoring, the scanning frequency per sensor, and individual scanning devices. Croatian and worldwide datasets were compared with worldwide data. The number of scans divided by the period of sensor operation yielded the scanning frequency for each sensor. Calculating the mean scan rate of all sensors and then finding the cumulative frequency distribution and summary metrics (mean, median, and interquartile range (IQR)) were used to determine the scanning frequency per reader.

Data analysis was performed by bins according to scanning frequency and by bins according to TIR. Each bin was compared successively.

### 2.3. Outcomes

The primary outcome was the association between scanning frequency and glucose metrics parameters (glucose management indicator, GMI; TIR; time above range, TAR; time below range, TBR; standard deviation of glucose, SD; coefficient of variation, %CV). The additional outcome was to determine the optimal number of scans per day necessary to acquire adequate glycemic regulation by grouping data in bins according to TIR, thus avoiding high-frequency outliners. In addition, in both types of analysis, each bin was compared successively. Croatian glucose parameters were also compared with worldwide data.

### 2.4. Statistical Analysis

The frequency of scanning per day and per hour was gathered to analyze scanning trends. Furthermore, glycemic analysis was carried out using the available data. Each sensor had to have at least 120 operational hours to be included in the analysis, ensuring trustworthy glucose control metrics. All data from the same reader’s sensors were aggregated and analyzed.

Mean glucose, TIR (defined as glucose between 3.9 and 10 mmol/L), TAR (>10 mmol/L and >13.9 mmol/L), and TBR (<3.9 mmol/L, <3.0 mmol/L and <2.5 mmol/L) were all employed as glycemia measurements [13]. Additional data such as time spent in very high glucose and very low ranges defined as >250 mg/dL (13.9 mmol/L) and <54 mg/dL (<3.0 mmol/L) were also evaluated. A formula based on the ADAG study was used for calculating GMI [14].

For each 5% of available readers, the cumulative frequency of scan rates and the mean GMI were computed to stratify the readers into 10 equally sized groups (bins) according to scanning frequency and TIR, and descriptive statistics were calculated. Scanning patterns were evaluated based on the frequency distribution of scans by an hour of the day.

The database was analyzed by structured query language routines and further summarized by KNIME (www.knime.org). Data were further analyzed using the statistical software SPSS (IBM, V 25.0), as well as online calculator statpages.info.

The means of glucose metrics (daily scans, average glucose, SD, glucose %CV, TIR, TAR, TBR) were compared between the highest and lowest TIR and GMI bins using independent samples *t*-tests.

Statistical comparisons across the groups were performed by one-way analysis of variance using Tukey’s HSD post hoc tests to avoid false significant results (type II error) due to multiple comparisons. Statistical significance of *p* < 0.05 was used.

## 3. Results

**Figure 1:**
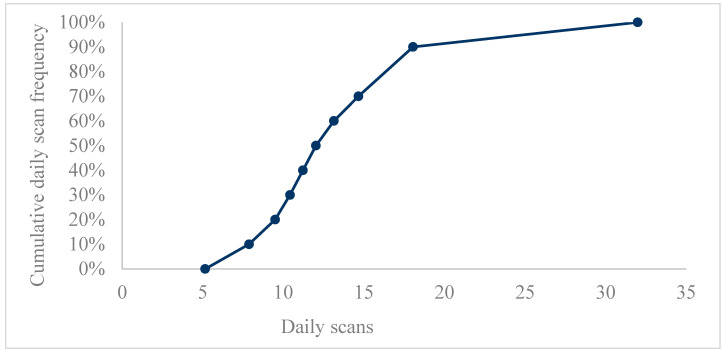
The cumulative distribution of daily scans by readers.

FSL users in Croatia performed on average 13 scans per day (M = 13.4 ± 7.4), with daily scans by readers shown in Table 1. The cumulative distribution of daily scans by readers is presented in Figure 1.

### 3.1. Data Analysis According to Bins of Scanning Frequency

Glycemic parameters according to bins of scanning frequency in the Croatian population are summarized in Table 2. A series of one-way ANOVA was calculated, with Tukey’s post hoc tests showing significant differences between each successive bin of scanning frequency for every parameter measured. There was a significant difference in scanning frequency (F(9,6210) = 1834.313, *p* < 0.001) between bins—the scanning frequency increased in bin 2 compared to bin 1, as well as in bin 3 compared to bin 2 (*p* < 0.001). Bin 4 had an increased frequency compared to bin 3 at the *p* = 0.006 level. There was no difference from bins 4 to 6 (*p* = 0.037), but bins 8, 9, and 10 each showed a significant increase in scanning frequency (all *p* < 0.001).

A significant difference in average glucose levels (mmol/L) (F(9,6210) = 39.381, *p* < 0.001) between bins was present—the average glucose decreased in bin 2 compared to bin 1 (*p* < 0.001), but not in bin 3 compared to bin 2 (*p* = 0.536), while bin 4 showed a decrease compared to bin 3 at *p* = 0.042. There was no significant successive decrease in glucose levels from readers in bins 5 through 10 (all *p* > 0.05).

A similar result was found for GMI (%), where the difference was significant between bins (F(9,6210) = 39.170, *p* < 0.001)—GMI decreased in bin 2 compared to bin 1 (*p* < 0.001), but not in bin 3 compared to bin 2 (*p* = 0.543). Bin 4 had a decrease in GMI at level *p* = 0.046. Also, no difference was noted in bins 5 through 10 (*p* > 0.05 for all). Regarding TIR, a significant difference (F(9,6210) = 48.077, *p* < 0.001) between bins was observed—TIR (%) increased in bin 2 compared to bin 1 (*p* < 0.010), but not in bin 3 compared to bin 2 (*p* = 0.807). Bin 4 had an increased percentage of TIR at *p* = 0.032 compared to bin 3. Readers in bins 5 through bin 8 did not show a significant increase in TIR (all *p* > 0.05) but, in bin 9 compared to 8, there was a significance at a level of *p* = 0.008. However, no significant increase in bin 10 compared to bin 9 was shown (*p* = 0.994).

The mean glucose SD (F(9,6210) = 57.870, *p* < 0.001) was also significantly different between bins—the average glucose variation decreased in bin 2 compared to bin 1 (*p* < 0.001), but not in bin 3 compared to bin 2 (*p* = 0.173), while bin 4 showed a decrease compared to bin 3 with a significance level of *p* = 0.048. No difference was observed in bins 5 through 8 (all *p* > 0.05), but there was a significant decrease in bin 9 compared to bin 8 *p* = 0.029, and further in bin 10 at the level *p* = 0.048.

As for the %CV, a significant difference (F(9,6210) = 35.333, *p* < 0.001) between bins existed—but the post hoc test demonstrated that the only significant decrease appeared successively in bin 10 compared to bin 9 (<0.001).

Bin 10 had a lower glucose level, GMI, mean glucose SD, and %CV compared to bin 1 (*p* < 0.001 for all), as well as a higher daily scanning frequency and TIR compared to bin 1 (*p* < 0.001).

Data regarding time above and time below range indices are summarized in Table 3. There was a statistically significant difference in TBR (<2.5 mmol/L) (F(9,6210) = 8.369, *p* < 0.001), TBR (<3.0 mmol/L) (F(9,6210) = 9.125, *p* < 0.001), and TBR (<3.9 mmol/L) (F(9,6210) = 4.302, *p* < 0.001) between bins, but not successively—only bin 9 differs significantly compared to bin 10 (*p* < 0.001). Also, bin 10 had a significantly lower TBR compared to bin 1 (*p* < 0.001).

With respect to TAR (>10.0 mmol/L), (F(9,6210) = 37.599, *p* < 0.001) and TAR (>13.9 mmol/L), (F(9,6210) = 50.746, *p* < 0.001), a significant difference was present between bins—TAR (%) decreased in bin 2 compared to bin 1 (*p* < 0.001), but not in bin 3 compared to bin 2 (*p* = 0.891). Bin 4 had a decreased percentage of TAR at *p* = 0.035 compared to bin 3. Readers in bins 5 through bin 8 did not show a significant change in TAR (>10.0 mmol/L) (*p* > 0.05 for all), but there was a meaningful change between bin 9 and 8 at *p* = 0.038. There was no significant decrease in bin 10 (*p* = 0.994). As for the TAR (>13.9 mmol/L), there was no change in bins 5 through bin 10 (all *p* > 0.05).

Bin 10 had lower daily TAR > 13.9 and TAR > 10.0 mmol/L compared to bin 1 (*p* < 0.001).

**Figure 2:**
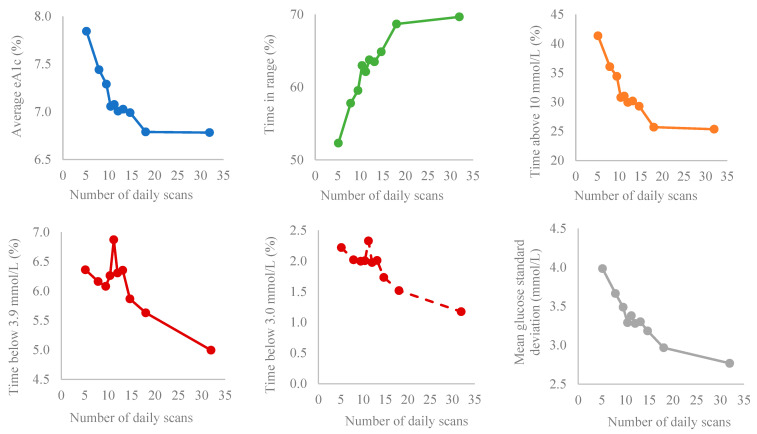
Relationship between frequency of daily scans and glucometrics according to bins of scanning frequency.

The relationship between the frequency of daily scans and glucometrics is summarized in Figure 2.

### 3.2. Data Analysis According to Bins of TIR

A series of one-way ANOVA was calculated, with Tukey’s post hoc tests showing statistically significant differences between each successive bin of TIR for each parameter measured (Table 4). There was a significant difference in scanning frequency (F(9,6210) = 28.739, *p* < 0.001) between all bins. Still, there were no successive differences between bins up to bin 9, which had a statistically higher scanning frequency compared to bin 8 (*p* = 0.002). A further increase was seen in bin 10 compared to bin 9 (*p* < 0.001).

Regarding the average glucose level (mmol/L) and GMI, there was a significant difference between bins (F(9,6210) = 2575.564, *p* < 0.001, F(9,6210) = 2575.884, *p* < 0.001 respectively)—the average glucose and GMI decreased between all successive bins (all *p* < 0.001).

Accordingly, a significant difference in TIR (F(9,6210) = 21786.077, *p* < 0.001) between bins was present with an increase in TIR from bin 1 to bin 10 (all *p* < 0.001).

The %CV (F(9,6210) = 608.123, *p* < 0.001) also significantly differed between bins—there was a significant decrease in bin 2 compared to bin 1 (*p* < 0.001), but not in bin 3 compared to bin 2 (*p* = 0.999), nor in bin 4 compared to bin 3 (*p* = 0.999). Then, a decrease in bin 5 compared to bin 4 at the level *p* = 0.095 was detected, as well as between bin 6 and bin 5 (*p* = 0.095). Afterward, each bin showed a significant decrease in %CV (all *p* < 0.001). On the other hand, the average glucose deviation decreased in each successive bin from bin 1 to bin 10 (all *p* < 0.001).

Bin 10 had a significantly lower GMI, average glucose level, average glucose variation and %CV compared to bin 1 (*p* < 0.001 for all), and higher TIR and scanning frequency (*p* < 0.001 for all).

Subsequently, hypoglycemia and hyperglycemia indices were calculated in all bins of TIR (Table 5). There was a statistically significant difference in TBR (<2.5 mmol/L) (F(9,6210) = 50.332, *p* < 0.001) between bins, but not successively—only bin 2 differed compared to bin 1 (*p* < 0.001), and then no significant differences were found up to bin 7 that were significantly lower than bin 6 at the level *p* = 0.027, as well as bin 8 compared to bin 7 (*p* = 0.027). Bin 9 further decreased (*p* < 0.001), but there was no further decrease in TBR in bin 10.

Although a significant difference in TBR (<3.0 mmol/L) (F(9,6210) = 71.974, *p* < 0.001) between bins was observed, only bin 2 differed significantly compared to bin 1 (*p* < 0.001), and then no significant differences were found up to bin 7 that were significantly lower than bin 6 at the level *p* = 0.036. There were further successive decreases in bins 8 (*p* = 0.004), 9 (*p* < 0.001), and 10 (*p* = 0.004).

TBR (<3.9 mmol/L) also differed between bins (F(9,6210) = 91.615, *p* < 0.001)—bin 2 differed significantly compared to bin 1 (*p* < 0.001), as well as bin 3 compared to bin 2 (*p* = 0.010). There were no further decreases until bin 9, which was significantly lower than in bin 8 (*p* < 0.001), with an additional decrease in bin 10 (*p* < 0.001).

Bin 10 had a significantly lower TBR < 3.0 mmol/L and TBR < 2.5 mmol/L than bin 1 (*p* < 0.001 for all), yet there was no difference in TBR < 3.9 mmol/L.

Regarding hyperglycemia indices, there was a significant difference in TAR (>10.0 mmol/L) and TAR (>13.9 mmol/L), (F(9,6210) = 5503.852, *p* < 0.001; F(9,6210) = 3697.117, *p* < 0.001, respectively) between bins—both parameters decreased in each successive bin from bin 1 to bin 10 (all *p* < 0.001). Bin 10 had significantly lower daily TAR > 13.9 mmol/L and TAR > 10.0 mmol/L compared to bin 1 (*p* < 0.001 for all).

### 3.3. Croatian and Worldwide Data Analysis According to Bins of TIR

**Figure 3:**
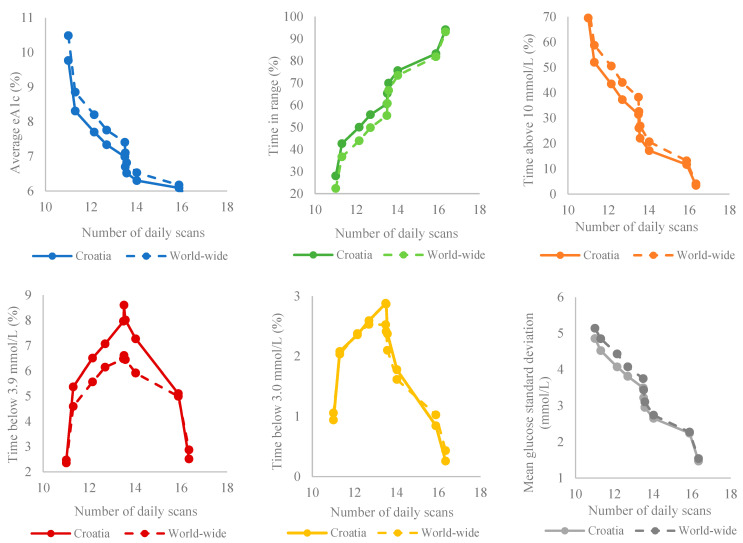
Croatian and worldwide glucometrics according to bins of TIR.

A comparison between Croatia and the worldwide data is presented in Figure 3.

A series of T-tests were calculated, comparing means for Croatia and worldwide according to bins of TIR at each point of measurement (due to multiple comparisons, a level of *p* < 0.001 was considered significant). Except for the last bin, Croatia had significantly higher levels of daily scan frequency and lower levels of GMI than those worldwide. In bins 2–8, TBR (<3.9 mmol/L) was significantly higher in Croatia, whereas TIR was higher in all bins worldwide at a significant level; in addition, TAR was lower compared to Croatian data. The same applies to glucose variation, which was lower in all bins in Croatia apart from bin 9.

## 4. Discussion

Regularly monitoring glucose levels is essential for reaching and maintaining glycemic targets, which translates to outcomes, leading to a reduced risk of acute adverse events (hypoglycemia and hyperglycemia), improved quality of life, and, in the long run, fewer micro- and macrovascular diabetes-related complications [15]. Recent research demonstrated that isCGM with FSL in real world-conditions with a scanning frequency of >20 times per day is associated with an eA1C level of around 7.0% (53 mmol/mol), which is, for the majority of patients, the target A1C [16,17]. Our patients performed on average 13.4 scans per day and achieved a GMI of 7.03% (53 mmol/mol).

In several RW studies from other countries that included a large number of subjects, it was shown that an increasing frequency of scans was associated with a decreasing time spent either in hypoglycemia or in hyperglycemia, suggesting a clear association between the increasing number of scans with better glucose management [16,17,18,19,20,21].

Still, in our study, a comparison between each consecutive bin per scanning frequency allowed for a slightly different perspective.

According to our results, benefits in terms of GMI reduction were achieved with an additional 1.5 daily scans, while a further increase in the number of scans did not offer any clinically meaningful benefits to patients. In our patient cohort, those with the lowest scanning frequency (5.1 ± 1 scans per day) had a GMI of 7.84 ± 1.70% and, with an increase in scanning of 2.7 scans per day, achieved improvements in GMI reduction, meaning there was no improvement between bin 5 and 10. This similarly applies to average glucose levels and TIR, with one exception: there was a significant difference in TIR comparing bins 8 to 9.

Still, the %CV and mean glucose SD completely depended on the frequency of scanning; unlike the previously mentioned parameters, the higher the frequency of scanning, the lower the %CV. These data correspond with the fact that the TBR defined at lower glycemic values (<2.5 mmol/L, <3.0 mmol/L) also depended on the scan frequency, i.e., a difference was observed between consecutive bins even at the highest scan frequencies per day, while the same was not shown for the TBR defined as a glucose level below 3.9 mmol/L. Regarding TAR, a difference was observed when the upper glycemic level was set above 10 mmol/L between bins 9 and 10, but not between bins 5 and 9. However, when the limit was set above 13 mmol/L, this difference disappeared and there was no marked improvement from bin 5 to bin 10. This was completely expected, given that hypo- and hyperglycemia contribute the most to the %CV and glucovariability, especially in the extreme glucose ranges [13].

It seems that the scanning frequency sufficient for achieving meaningful improvements in glycemic regulation would be in bin 5 (11.2), while the additional benefit in terms of decreasing severe hypoglycemia as well as the reduction in the %CV could be achieved with an increasing number of scans up to bin 10 (31 ± 0.98). Additionally, an increasing scanning frequency from bin 8 to bin 9 (from 14.8 ± 0.5 to 18 ± 1.4) could be useful in improving TIR and reducing TAR (>10 mmol/L). The arrival of the FSL system with an alarm function would surely further assist in the avoidance of hypo- and hyperglycemia, without the need for a significant increase in the number of scans [22]. To confirm our results, an additional analysis was performed according to bins of TIR. From the standpoint of everyday clinical practice, it is important to advise patients on how to best utilize technology while at the same time not slipping into the over-use category, which might lead to suboptimal decision making in terms of glucose management. The international consensus recommends that individuals with T1DM should spend >70% of their time within the target range (3.9–10.0 mmol/L [70–180 mg/dL]) and strive to achieve <4% below the target range (<3.9 mmol/L [<70 mg/dL]) and <25% above the range (>10.0 mmol/L [180 mg/dL]), with %CV ≤36% [13]. In our study, FSL users who spent the most time within the range (94.09 ± 3.49%) achieved that with 16.3 ± 10,5 daily scans, with a %CV of 22.97 ± 4.94%, confirming previously published data demonstrating that users performing an average of 16.3 daily glucose scans achieved meaningful improvement in glucose metrics [16], avoiding the detrimental effects on the quality of life resulting from the burden due to excessive scanning on a daily basis.

As for all glucometric parameters in this type of analysis, improvement was shown between each successive bin of TIR, especially in bins with a higher scanning frequency, as opposed to the previous analysis performed only according to scanning frequency. This discrepancy can be explained by a difference in the number of scans per day, which was 16.3 ± 10.5 in bin 10 according to TIR-based analysis as opposed to 31.9 ± 13.5 in bin 10 according to the analysis based on scan frequency.

When comparing the global data and the Croatian data according to bins of TIR, it is noticeable that the Croatian users scan more frequently compared to the rest of the world, and therefore have lower GMI values, except in the last bin, where the difference in frequency is the smallest (16.3 ± 10.5 vs. 15.3 ± 13.3). It is interesting to note that a higher percentage of TBR was recorded in Croatia compared to the world, which can be attributed to a higher frequency of scanning; however, this difference is again lost in bins 9 and 10, where the difference in the number of scans is minimal. For example, in a study by Lameijer et al. [17], Dutch isCGM users had higher GMI values compared to the rest of the world whereas Polish data demonstrated better glycemic regulation than that worldwide, similar to Croatian data [20]. This could be attributed to different reimbursement policies between countries influencing the population included in the analysis; for instance, only T1DM users or all patients using intensive insulin therapy regardless of the type of diabetes. Moreover, indications for isCGM prescription could differ by region or county, encompassing proven hypoglycemic incidents and/or poor glycemic regulation defined as A1C > 7% (53 mmol/mol).

This study has some limitations. All data were anonymous; therefore, patients’ characteristics that could possibly influence final outcomes such as age, duration of diabetes, type of insulin therapy, and dietary habits, as well as exercise patterns, were unavailable. However, in the management of T1DM, exercise represents one of the greatest challenges for optimal blood glucose maintenance, possibly requiring a higher frequency of scans before, during, and after the exercise. Given the importance and influence on glycemic stability, exercise should be implemented and considered in future studies, as it guides the development of glucose monitoring systems and carbohydrate/insulin estimation algorithms [23,24,25].

## 5. Conclusions

In previously published studies, analysis by deciles of the scan frequency comparing only bins 1 to 10 was performed, calling attention to outliers with a high scan frequency and creating the misconception that FSL users must scan extremely frequently to achieve a high TIR. In the present study, we have performed analysis by deciles of scanning frequency and TIR and also a comparison between each successive bin to avoid attention to outliers with a high scan frequency. Therefore, according to our results, isCGM systems used in everyday clinical practice can contribute to a significant improvement in glucoregulation, i.e., achieving good glycemic regulation comparable to rtCGM systems with an optimal number of 16.3 scans per day [26]. Also, our analysis was performed using isCGM FSL without an alarm function predicting low and high glucose as opposed to rtCGM systems. New improved isCGM systems equipped with predictive low and high alarms will further improve the possibility of reaching glycemic goals without a need for an excessive scanning frequency.

## Figures and Tables

**Table 1:** Average scanning frequency per day in Croatia.

	Mean	Std. Deviation	Minimum	Maximum	Percentiles		
					25th	50th (Median)	75th
Daily Scans Mean	13.40073	7.438523	5.149	31.981	9.08825	11.62062	15.51041

**Table 2:** Mean values for scanning frequency and glucose parameters for all 10 bins according to scanning frequency.

Groups (Bins by Deciles)	Scan Rate (Scans/Day)		Average Glucose (mmol/L)		GMI (%)		Time in Range (%)		Glucose Standard Deviation (mmol/L)		CV (%)	
	Mean	SD	Mean	SD	Mean	SD	Mean	SD	Mean	SD	Mean	SD
1	5.15	1.07	9.91	2.71	7.84	1.70	52.31	21.59	3.98	1.49	39.74	10.24
2	7.87 *	0.63	9.27 *	2.26	7.44 *	1.42	57.79 *	20.05	3.66 *	1.31	38.93	9.41
3	9.49 *	0.31	9.03	1.98	7.29	1.24	59.53	17.93	3.49	1.09	38.30	7.98
4	10.42	0.24	8.66 *	1.85	7.06 *	1.16	62.96 *	17.11	3.29 *	0.99	37.71	7.94
5	11.22	0.22	8.69	1.89	7.08	1.18	62.11	16.62	3.38	0.96	38.78	7.16
6	12.02	0.28	8.58	1.69	7.01	1.06	63.75	16.12	3.28	0.92	38.01	7.13
7	13.14	0.37	8.61	1.81	7.03	1.14	63.47	16.73	3.30	1.00	38.03	7.82
8	14.66 *	0.59	8.55	1.87	6.99	1.17	64.84	17.58	3.18	1.02	36.91	7.94
9	18.05 *	1.42	8.23	1.88	6.79	1.18	68.67 *	18.27	2.97 *	1.07	35.36	8.53
10	31.98 *	13.53	8.22	2.12	6.78	1.33	69.65	19.90	2.77 *	1.11	32.94	8.78

* *p* < 0.05 compared to the previous bin; GMI, glucose management indicator; CV, coefficient of variation. The average confidence interval ranges from ±0.02 to ±0.04 for all variables above, except for CV, where 95% CI is ±0.16, and for TIR, with an average 95% CI of ±0.36. Only TIR shows a larger interval for mean estimation (meaning that, with 95% certainty, we can assume that the mean of the population would range ±0.36 below and above the measured mean of the sample).

**Table 3:** Time below and time above range indices in all 10 bins according to scanning frequency.

Groups (Bins by Deciles)	Time Below Range (%)						Time Above Range (%)			
	<2.5 mmol/L		<3.0 mmol/L		<3.9 mmol/L		>10.0 mmol/L		>13.9 mmol/L	
	Mean	SD	Mean	SD	Mean	SD	Mean	SD	Mean	SD
1	1.06	2.24	2.22	3.57	6.36	7.54	41.33	22.94	19.11	17.53
2	0.94	1.84	2.02	2.85	6.16	5.62	36.05 *	20.97	14.67	14.51
3	0.92	1.51	2.00	2.66	6.08	5.58	34.39	19.30	12.24	12.40
4	0.92	1.59	2.00	2.69	6.26	5.53	30.77 *	18.42	9.89	10.60
5	1.11	1.90	2.33	3.15	6.87	6.15	31.02	18.25	10.25	11.63
6	0.91	1.67	1.98	2.78	6.31	5.58	29.94	17.38	9.21	9.89
7	0.95	1.72	2.01	2.77	6.35	5.86	30.18	18.11	9.78	10.57
8	0.78	1.58	1.73	2.68	5.87	5.73	29.29	18.84	9.30	11.04
9	0.64	1.33	1.52	2.47	5.63	6.12	25.70 *	18.93	7.86	10.19
10	0.46 *	1.12	1.17 *	2.31	4.99	6.73	25.36	21.04	7.89	11.75

* *p* < 0.05 compared to the previous bin. The average confidence interval ranges from ±0.03 to ±0.12 for the time below range, while, for the time above range, 95% CI is ±0.24 to ±0.38.

**Table 4:** Mean values for scanning frequency and glucose parameters for all 10 TIR-based bins.

TIR Bins (Deciles)	Scan Rate (Scans/Day)		Average Glucose (mmol/L)		GMI		TIR (%)		Glucose Standard Deviation (mmol/L)		CV (%)	
	Mean	SD	Mean	SD	Mean	SD	Mean	SD	Mean	SD	Mean	SD
1	11.01	6.98	12.98	1.75	9.77	1.10	28.01	8.32	4.86	1.18	37.58	8.52
2	11.31	6.19	10.65 *	0.83	8.31 *	0.52	42.61 *	2.63	4.52 *	0.82	42.56	7.83
3	12.15	7.44	9.68 *	0.85	7.70 *	0.53	50.04 *	1.78	4.07 *	0.63	42.29	7.32
4	12.70	7.49	9.10 *	0.76	7.34 *	0.48	55.68 *	1.44	3.82 *	0.48	42.29	6.77
5	13.50	6.98	8.55 *	0.84	6.99 *	0.53	60.60 *	1.37	3.49 *	0.44	41.25	6.36
6	13.52	7.15	8.09 *	0.90	6.70 *	0.56	65.33 *	1.29	3.22 *	0.36	40.21	5.55
7	13.58	6.95	7.80 *	0.86	6.52 *	0.54	69.95 *	1.45	2.96 *	0.33	38.22 *	4.95
8	14.03	7.70	7.45 *	0.84	6.30 *	0.53	75.58 *	1.88	2.65 *	0.34	35.77 *	4.43
9	15.88 *	11.89	7.11 *	0.76	6.09 *	0.48	83.25 *	2.57	2.24 *	0.32	31.57 *	3.96
10	16.35 *	10.57	6.32 *	0.75	5.59 *	0.47	94.09 *	3.49	1.47 *	0.40	22.97 *	4.94

* *p* < 0.05 compared to the previous bin; TIR, time in range; GMI, glucose management indicator; CV, coefficient of variation. The average confidence interval ranges from ±0.01 to ±0.16 for all variables.

**Table 5:** Time below and time above range indices in all 10 TIR-based bins.

Groups (Deciles)	Time below Range (%)						Time above Range (%)			
	<2.5 mmol/L		<3.0 mmol/L		<3.9 mmol/L		>10.0 mmol/L		>13.9 mmol/L	
	Mean	SD	Mean	SD	Mean	SD	Mean	SD	Mean	SD
1	0.47	1.14	0.94	2.02	2.47	4.37	69.52	10.20	39.40 *	13.23
2	1.10 *	2.31	2.08 *	3.31	5.37 *	5.90	52.02 *	6.61	22.79 *	5.51
3	1.12	2.09	2.36	3.38	6.50 *	6.69	43.46 *	6.98	15.49 *	4.00
4	1.23	1.86	2.59	3.13	7.07	5.91	37.25 *	6.22	11.56 *	3.24
5	1.42	2.15	2.88	3.42	7.97	6.62	31.44 *	6.73	7.97 *	2.79
6	1.31	1.97	2.87	3.36	8.60	6.90	26.07 *	7.07	5.53 *	2.31
7	1.00 *	1.48	2.37 *	2.74	8.02	6.48	22.04 *	6.65	3.85 *	1.88
8	0.69 *	1.03	1.78 *	2.08	7.27	5.91	17.15 *	6.06	2.37 *	1.45
9	0.27 *	0.49	0.85 *	1.13	5.10 *	4.45	11.64 *	4.94	1.09 *	0.87
10	0.08	0.26	0.26 *	0.49	2.51 *	2.46	3.40 *	3.11	0.12 *	0.22

* *p* < 0.05 compared to the previous bin. The average confidence interval ranges from ±0.03 to ±0.13 for all variables.

## Data Availability

The data presented in this study are available on request from the corresponding author. The data are not publicly available due to privacy of patients.
